# Guide to detecting epidermal growth factor receptor (*EGFR*) mutations in ctDNA of patients with advanced non-small-cell lung cancer

**DOI:** 10.18632/oncotarget.13915

**Published:** 2016-12-12

**Authors:** Nicola Normanno, Marc G. Denis, Kenneth S. Thress, Marianne Ratcliffe, Martin Reck

**Affiliations:** ^1^ Cell Biology and Biotherapy Unit, Istituto Nazionale Tumori Fondazione Giovanni Pascale, IRCCS, Napoli, Italy; ^2^ Department of Biochemistry, Nantes University Hospital, Nantes, France; ^3^ AstraZeneca, Waltham, MA, USA; ^4^ AstraZeneca, Macclesfield, UK; ^5^ Department of Thoracic Oncology, LungenClinic Grosshansdorf, Grosshansdorf, Airway Research Center North (ARCN), Member of the German Centre for Lung Research (DZL), Germany

**Keywords:** ctDNA, NSCLC, EGFR, T790M

## Abstract

Cancer treatment is evolving towards therapies targeted at specific molecular abnormalities that drive tumor growth. Consequently, to determine which patients are eligible, accurate assessment of molecular aberrations within tumors is required. Obtaining sufficient tumor tissue for molecular testing can present challenges; therefore, circulating free tumor-derived DNA (ctDNA) found in blood plasma has been proposed as an alternative source of tumor DNA. The diagnostic utility of ctDNA for the detection of epidermal growth factor receptor (*EGFR*) mutations harbored in tumors of patients with advanced non-small-cell lung cancer (NSCLC) is supported by the results of several large studies/meta-analyses. However, recent real-world studies suggest that the performance of ctDNA testing varies between geographic regions/laboratories, demonstrating the need for standardized guidance. In this review, we outline recommendations for obtaining an accurate result using ctDNA, relating to pre-analytical plasma processing, ctDNA extraction, and appropriate *EGFR* mutation detection methods, based on clinical trial results. We conclude that there are several advantages associated with ctDNA, including the potential for repeated sampling particularly following progression after first-line tyrosine kinase inhibitor (TKI) therapy, as TKIs targeting resistance mutations (eg T790M) are now approved for use in the USA/EU/Japan (at time of writing). However, evidence suggests that ctDNA does not allow detection of *EGFR* mutations in all patients with known mutation-positive NSCLC. Therefore, although tumor tissue should be the first sample choice for *EGFR* testing at diagnosis, ctDNA is a promising alternative diagnostic approach.

## INTRODUCTION

The treatment of cancer is evolving from toxic, broad chemotherapeutic approaches to therapies targeted towards specific molecular abnormalities that drive tumor growth. In order to continue this evolution, robust and accurate assessment of molecular aberrations within tumors is required to determine which patients are suitable for these targeted therapeutics, and, in turn, which therapies are appropriate in different settings. Generally, molecular testing has been performed using tumor tissue obtained by surgery or biopsy. Adequate tumor (tissue or cytology) samples taken in a suitable form are clinically important for a complete pathological diagnosis including tumor typing and subtyping, and analysis of predictive markers [1]. It can be challenging to obtain sufficient tumor tissue for molecular testing (particularly where biopsy samples are small or are prioritized for disease diagnosis) and invasive biopsy procedures may present with too great a health risk for some patients. Indeed, 27–31% of patients with non-small-cell lung cancer (NSCLC) may be unable to provide a biopsy sample suitable for epidermal growth factor receptor (*EGFR*) mutation analysis at diagnosis [2] or following disease progression. Analysis of circulating free tumor-derived DNA (ctDNA) has been proposed as an alternative, minimally invasive method for the detection of *EGFR* mutations [3, 4].

Genetic mutations in DNA found in the serum of patients with NSCLC were first observed in 1998 [5]. In 2006, the potential clinical value of ctDNA was uncovered when *EGFR* mutations detected in serum were associated with response to gefitinib in a small, retrospective study [6]. As the link between *EGFR* mutations in NSCLC and response to EGFR tyrosine kinase inhibitor (EGFR TKI) therapy was further elucidated [7], the clinical utility of ctDNA was more robustly investigated *via* preplanned analyses in large-scale studies. The presence of *EGFR* mutations in ctDNA was shown to predict response to the EGFR TKIs gefitinib and erlotinib in the first-line setting [8, 9]. Moreover, objective response rates and progression-free survival were found to be similar in patients with *EGFR* mutations detected in their ctDNA and in those who were identified as *EGFR* mutation-positive in their tumor sample [10]. Detection of resistance mutations such as T790M and C797S in the plasma of patients with NSCLC who progress on EGFR TKIs also has promising utility in identifying which patients are suitable for subsequent therapies, both following initial diagnosis and following disease progression with first-line TKI therapy [11–14].

The process by which tumor DNA enters the bloodstream is not fully understood. Diehl et al reported a correlation between the amount of mutant ctDNA in colorectal cancer and the invasiveness of the tumor [15]. This finding, along with the highly fragmented nature of ctDNA, led the authors to propose that the DNA came from necrotic neoplastic cells that had been phagocytized by macrophages [15]. It has also been proposed that ctDNA is released by apoptotic cells [16, 17], based on the observation that ctDNA forms a ladder pattern during electrophoresis, which is reminiscent of that produced by apoptosis [18]. Another possible source of ctDNA is the breakdown of circulating tumor cells; however, in patients with a range of malignancies (excluding lung), levels of ctDNA have been observed to be higher than that of circulating tumor cells, suggesting that these cells are not the source of ctDNA [19]. Finally, it has been suggested that tumor cells may actively secrete DNA fragments, as has been observed for lymphocytes and seen recently in patients with NSCLC *via* extracellular vesicles (exosomes) [20, 21].

Meta-analyses have indicated that detection of *EGFR* mutations using ctDNA may be effective for diagnostic purposes [22–24]. However, results from individual studies are variable, with many indicating that detection of *EGFR* mutations in ctDNA is more difficult in plasma samples than in tumor tissue, with an average sensitivity of 65–70% (see Table 1 for definition of concordance parameters) [25]. Indeed, ctDNA is frequently highly diluted and, therefore, highly sensitive techniques are required in order to detect somatic mutations in blood samples. Recent real-world studies [26, 27] have shown that the performance of ctDNA testing varies significantly between different geographic regions and different laboratories, demonstrating the need for standardized and well-validated ctDNA methodology. With recommendations based upon methods for ctDNA mutation analysis that have been employed in clinical trials, in this review, we look at the key steps required to ensure accurate ctDNA test results and propose options to optimize sensitivity.

**Table 1:** Definitions of specificity, sensitivity, positive predictive value, and negative predictive value

True positive	Patient's tumor carries the mutation and patient tests positive for the mutation
True negative	Patient's tumor does not carry the mutation and patient tests negative for the mutation
False positive	Patient's tumor does not have the mutation, yet tests positive for the mutation
False negative	Patient's tumor carries the mutation, yet tests negative for the mutation
Sensitivity	True positive / (true positive + false negative)
Specificity	True negative / (true negative + false positive)
Positive predictive value	True positive / (true positive + false positive)
Negative predictive value	True negative / (true negative + false negative)

## PRE-ANALYTICAL FACTORS

### Plasma *versus* serum

In order to measure any biomarker, optimization of sample processing, handling, and storage is needed to ensure that the biomarker is neither degraded nor masked by other constituents of the sample. For example, it is important to reduce contamination of ctDNA samples by wild-type DNA originating from circulating leukocytes, which has the potential to mask the small (low ng/mL) quantities of tumor DNA in the sample. For this reason, plasma is preferable to serum because the clotting process in serum leads to the release of genomic DNA from white blood cells (reviewed by El Messaoudi et al 2013 [28]). Furthermore, a head-to-head comparison of analysis of plasma and serum ctDNA samples from the same patients demonstrated higher sensitivity with plasma *versus* serum [29], thus confirming the assertion that the use of plasma is preferable for *EGFR* mutation analysis of ctDNA. Common anticoagulants such as ethylenediaminetetraacetic acid (EDTA) and citrate are both suitable for processing of blood samples for ctDNA analysis [30], but it is recommended that heparin should be avoided since it may interfere with downstream polymerase chain reaction (PCR) applications [31].

### Plasma processing and storage

**Figure 1:**
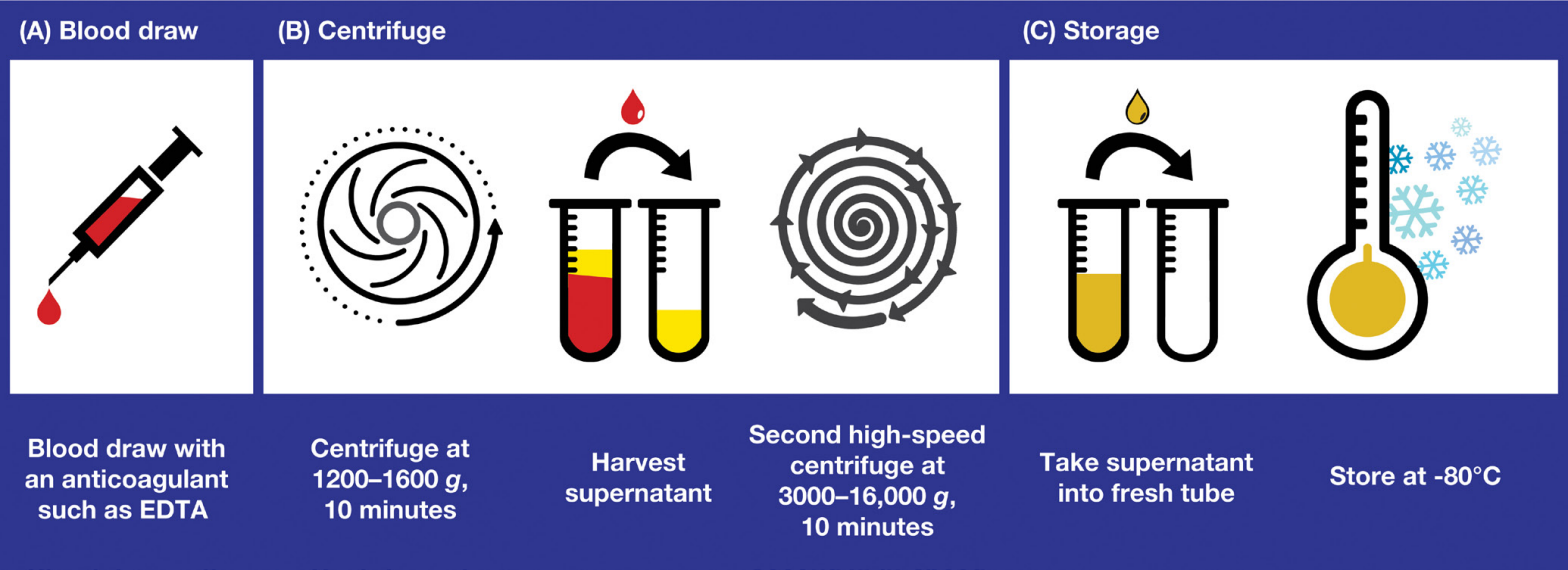
Obtaining plasma samples for ctDNA analysis: recommended workflow for plasma collection, processing, and storage The key pre-analytical steps involved in collecting plasma samples that are suitable for ctDNA analysis are shown. **A**. The timing from blood draw to plasma isolation is crucial for success of the test. EDTA tubes should be used only if the time from blood draw to delivery of the sample to the testing laboratory is within 4 hours. Alternatively, tubes containing specific fixatives that prevent the lysis of white blood cells should be used. **B**. Plasma is obtained by centrifugation of the blood sample at 1200–1600 *g* for 10 minutes and harvesting of the supernatant. A second, higher-speed centrifugation (eg 3000–16,000 *g*) in a microcentrifuge is recommended to remove residual cellular contamination and to generate a clean sample for further analysis. This second centrifuge may be performed either before or after freeze/thaw. **C**. Fresh plasma should be stored at -80°C in the long term (-20°C acceptable for ~1 month) and shipping, if required, should be on dry ice. Repeated freeze/thaw should be avoided. ctDNA, circulating free tumor-derived DNA; EDTA, ethylenediaminetetraacetic acid.

Aside from the *EGFR* mutation testing method selected for analysis of ctDNA samples, the quality of the test is also dependent on the laboratory successfully completing the pre-analytical steps. The recommended workflow for plasma collection, processing, and storage is summarized in Figure 1. The timing from blood draw to plasma isolation is crucial: blood should be processed within 4 hours of drawing, as an increase in total DNA (indicative of white cell degradation) occurs if blood remains unprocessed for longer periods. There is no difference in DNA yield between samples stored at room temperature or at 4°C within this 4-hour time frame [28]. If processing within 4 hours of blood draw is possible, then EDTA tubes should be used. In clinical practice however, processing an individual blood sample to obtain plasma within a 4-hour time frame may be challenging. An alternative option is to stabilize blood in the collection tube, using specific fixatives that allow the blood to be processed later at a more convenient time. Several commercial options are available (eg cell-free DNA BCT^®^ [Streck, Omaha, NE, USA] and cfD^TM^ [Ariosa Diagnostics, San Jose, CA, USA]), which claim to stabilize blood for up to 7 days without compromising downstream DNA analysis. A comparison of ctDNA testing in samples from patients with advanced breast cancer (for mutations in four known drivers in breast cancer: *PIK3CA* exon 9 and 20, *ESR1* ligand binding domain, *AKT1*, and *ERBB2*) showed no differences in samples taken in Streck cell-free DNA BCT^®^ tubes that were processed after 48–72 hours compared with samples that were processed immediately [32]. Streck cell-free DNA BCT^®^ tubes have also been shown to prevent lysis and cellular release of genomic DNA from ctDNA samples of patients with metastatic breast cancer, compared with PAXgene tubes (PreAnalytiX, Feldbachstrasse, Hombrechtikon, Switzerland) [33]. Furthermore, blood samples from patients with melanoma stored in Streck cell-free DNA BCT^®^ tubes at room temperature were found to remain serviceable for ctDNA testing, even after long-term storage (up to 10 days at room temperature) [34].

Plasma is obtained by centrifugation of the blood sample at 1200–1600 *g* for (typically) 10 minutes and harvesting of the supernatant. This process does not remove all cellular contamination; therefore, a second, higher-speed centrifugation (eg 3000–16,000 *g*) in a microcentrifuge or filtration through a 0.2 µM filter is recommended to remove residual cellular contamination and to generate a clean sample for further analysis [35]. This second spin can be performed either before or after the freeze/thaw [36]. Fresh plasma should be stored at -80°C in the long-term (-20°C is acceptable for ~1 month), and shipping, if required, should be in dry ice to avoid sample degradation. Repeated freeze/thaw should be avoided, although 2–3 cycles is considered acceptable [28]. Long-term stability of DNA in plasma is best demonstrated at -80°C, a temperature at which DNA may be stable for up to 9 months [28]; however, median plasma DNA concentrations are at risk of significantly decreasing over time [37].

Plasma processing as outlined above is relatively straightforward. However, stabilization tubes, although more expensive, could be used in regions where infrastructure is poor or to support testing when samples cannot be processed locally, and instead need to be shipped to external expert laboratories for analysis.

### DNA extraction

Many methods exist for DNA extraction, including individual laboratory protocols, company in-house procedures, and several commercial kits. However, there is a lack of consensus on optimal ctDNA extraction methods, with guideline recommendations tending to focus on tissue/cytology samples for *EGFR* mutation testing [38]. It should not be assumed that extraction methods validated for tumor tissue or other matrices are suitable for ctDNA, which is fragmented and present at such low concentrations that specialized extraction protocols are required. Using the wrong extraction method can significantly reduce the ability to detect mutations in ctDNA. Although a number of kits for ctDNA extraction from different commercial sources are available, few comparative data are available. One study compared the ability of three methods specifically designed for extraction of ctDNA (QIAamp^®^ circulating nucleic acid kit [QIAGEN, Manchester, UK], polymer mediated enrichment (PME) free-circulating DNA extraction kit [Analytik Jena, Jena, Germany], and DSP virus/pathogen midi kit performed on QIAsymphony^®^ [QIAGEN, Hilden, Germany]) to detect *KRAS* mutations in plasma samples from patients with NSCLC (*n* = 10). All methods demonstrated successful DNA extraction, although the DNA yields differed significantly. As was found in earlier research [39], the different yields, (measured by quantitative PCR [qPCR]), may have been due to isolation of fragments of differing size distributions, in turn due to the varying nucleic acid size exclusion limits in a given kit [40]. In the large, multicenter, non-interventional, diagnostic ASSESS study – which investigated the utility of ctDNA for *EGFR* mutation testing in a real-world diagnostic setting (EU/Japan) – an initial plasma sensitivity of only 17% was seen in a subset of 92 patients using the QIAamp^®^ MinElute^®^ virus spin kit for DNA extraction (QIAGEN, Manchester, UK) and a peptide nucleic acid (PNA) clamp-based detection method. However, this DNA extraction kit can isolate high molecular weight DNA, but not fragmented DNA. Indeed, sensitivity increased to 51% when the same PNA clamp-based detection method was used on DNA extracted from a higher volume of plasma using the QIAamp^®^ circulating nucleic acid kit, which has been designed to isolate fragmented DNA; this highlights the importance of using a method specifically developed for ctDNA [27, 41]. The QIAamp^®^ circulating nucleic acid kit was used in the IFUM study, in which data from ctDNA analysis were associated with response to gefitinib [10]. Other kits may also be adequate for ctDNA extraction, but we are not currently aware of extensive data supporting their use for this purpose.

Since it is likely that only a limited number of DNA copies in plasma will have arisen from a tumor, the need for a large volume of plasma is important: 2 mL has been shown to yield accurate results using various methods [29], advocating this as an appropriate volume for use in clinical practice.

## MUTATION DETECTION METHODS

ctDNA is present in blood at low levels amidst a significant background of wild-type DNA; therefore, a sensitive mutation detection method with the ability to detect mutant alleles that comprise < 1% of the total DNA is required. However, highly sensitive assays are at risk of yielding false-positive results (Table 1) unless robustly validated, and so a trade-off must occur between the sensitivity and specificity of a chosen assay. Clinically, it may be better to favor an assay with high specificity, at the slight expense of sensitivity, in order to ensure that targeted therapies are administered appropriately [42, 43] (see Table 2 [8,9,27,29,43–60] for sensitivity and specificity of selected assays). The quality of *EGFR* mutation testing methods may only be warranted by participating in External Quality Assessment (EQA) schemes. EQA schemes for liquid biopsy for *EGFR* mutations are not currently available (at time of writing), but have been announced for 2017.

**Table 2:** Specificity and sensitivity of selected assays

Assay	Sensitivity, n/N (%)		Specificity, n/N (%)		Reference (no. of matched tissue/cytology and plasma samples)
	*EGFR* sensitizing mutations*	T790M mutations (after EGFR TKI progression)	*EGFR* sensitizing mutations*	T790M mutations (after EGFR TKI progression)	
BEAMing	186/244 (76.2)		N/A (95.8)		IMPRESS study [44](n=261)
	49/60 (81.7)	33/45 (73.3)	2/3 (66.7)	9/18 (50)	Karlovich et al 2016 [45](n=63)
	43/51 (84.3)	33/41 (80.5)	65/67 (97.0)	14/24 (58.3)	Thress et al 2015 [43](n=38)
Cycleave^®^	29/57 (50.9)		132/133 (99.2)		ASSESS study [46](n=190)
ddPCR	30/37 (81.1)		N/A (97.0)		Zhu et al 2015 [47](n=86)
DHPLC	188/269 (69.9)		445/553 (80.5)		Huang et al 2012 [48](n=822)
High-resolution melting	29/45 (66.4)		73/75 (97.3)		Jing et al 2013 [49](n=120)
Mass spectrometry genotyping	8/18 (44.4)		11/13 (84.6)		Brevet et al 2011 [50](n=31)
Mutation-based PCR-quenching probe Exon 19 L858R	21/47 (44.7) 2/23 (8.7)		23/23 (100) 47/47 (100)		Nakamura et al 2012 [51](n=39)
Mutant-enriched PCR	7/18 (38.9)		11/13 (84.6)		Brevet et al 2011 [50](n=31)
	16/45 (35.6)		63/66 (95.5)		Zhao et al 2013 [52](n=111)
NGS-based deep sequencing Exon 19 L858R	N/A (50.9) N/A (51.9)		N/A (98.0) N/A (94.1)		Uchida et al 2015 [53](n=288)
L858R	N/A (70.6)		N/A		Yao et al 2016 [54] (n=39)
PNA-LNA PCR clamp	15/29 (51.7)		61/62 (98.4)		ASSESS study [46](n=91)
	16/26 (61.5)		70/70 (100)		Pasquale et al 2015 [55](n=96)
	97/164 (59.1)		N/A		Rosell et al 2009 [56](n=164)
	58/109 (53.2)		N/A		Rosell et al 2012 [9](n=109)
PNA-PCR clamp with AS-APEX assay	32/32 (100)		4/5 (80.0)		Yam et al 2012 [57](n=37)
SARMS	22/51 (43.1)		35/35 (100)		IPASS study [58](n=86)
	27/40 (67.5)		46/46 (100)		Liu et al 2013 [59] (n=86)
SARMS-based QIAGEN therascreen^®^ EGFR RGQ PCR kit	16/22 (72.7)		115/116 (99.1)		ASSESS study [27](n=138)
	69/105 (65.7)		546/547 (99.8)		IFUM study [8](n=652)
	17/26 (65.4)		70/70 (100)		Pasquale et al 2015 [55](n=96)
	18/19 (94.7)		66/66 (100)		Vallée et al 2013 [29](n=85)
Roche cobas^®^ AS-PCR	43/51 (84.3)	30/41 (73.2)	65/67 (97.0)	16/24 (66.7)	Thress et al 2015 [43](n=72)
	17/28 (60.7)		162/168 (96.4)		Weber et al 2014 [60](n=196)
	55/75 (73.3)	21/33 (63.6)	24/24 (100)	61/62 (98.4)	Karlovich et al 2016 [45] (n=55)

* Exon 19 deletion/L858R mutation.

AS, allele-specific; BEAMing, beads, emulsions, amplification, and magnetics; ddPCR, digital droplet polymerase chain reaction; DHPLC, denaturing high-pressure liquid chromatography; EGFR, epidermal growth factor receptor; N/A, not available; NGS, next-generation sequencing; PCR, polymerase chain reaction; PNA-LNA, peptide nucleic acid-locked nucleic acid; RGQ, Rotor-Gene Q; SARMS, Scorpion amplified-refractory mutation system; TKI, tyrosine kinase inhibitor.

**Table 3:** Key considerations for successful detection of *EGFR* mutations in ctDNA of patients with advanced NSCLC

Pre-analytical factors	
Plasma versus serum	Plasma (vs serum) should be used for ctDNA mutation analysis
Plasma processing and storage	Blood should be processed to plasma within 4 hours of draw; alternatively, use of stabilization collection tubes containing fixatives should be considered to allow blood processing at a later time Plasma is obtained via centrifugation of the blood sample; a second, high-speed spin (before or after freeze/thaw [3000‒16,000 *g*]) in a microcentrifuge is necessary to generate clean samples for mutation analysis Fresh plasma should be stored at -20°C or -80°C (on dry ice for shipping), with long-term stability of DNA in plasma best demonstrated at -80°C
DNA extraction	Use of DNA extraction methods specifically developed for ctDNA – which is fragmented and only present at low concentrations – is essential
Mutation detections methods	
	Traditional methods (Sanger, pyrosequencing) are not suitable for ctDNA mutation analysis due to low sensitivity PCR methods that increase the proportion of mutant to wild-type DNA (mutant enriched-PCR, SARMS, PNA clamping) provide greater sensitivity than traditional sequencing methods Enhancements of traditional PCR (ddPCR, BEAMing) demonstrate increased sensitivity ddPCR and NGS enable quantification of mutant *EGFR* levels and may be used to monitor treatment response and disease progression Alternative methods (mass spectrometry genotyping, DHPLC, and high-resolution melting) represent potentially efficient and reliable methods for routine diagnostic use

BEAMing, beads, emulsions, amplification, and magnetics; ctDNA, circulating free tumor-derived DNA; ddPCR, digital droplet polymerase chain reaction; *EGFR*, epidermal growth factor receptor; DHPLC, denaturing high-pressure liquid chromatography; NGS, next-generation sequencing; NSCLC, non-small-cell lung cancer; PCR, polymerase chain reaction; SARMS, Scorpion amplified-refractory mutation system.

### PCR-based methods

Traditional methods such as Sanger sequencing (which are unable to detect mutant DNA present at levels < 10–20% of total DNA) or pyrosequencing, which has a sensitivity of approximately 5%, are not suitable for ctDNA mutation analysis because they can lead to a high proportion of false negatives (Table 1) [61]. To date, most ctDNA data have been generated with allele-specific qPCR-based methods adapted to enrich for mutant DNA; these methods are generally able to detect 1–5% of mutant DNA. Adaptations to improve sensitivity include the use of mutation-specific primers. For example, the amplified-refractory mutation system (ARMS) [62] combined with Scorpion molecules (SARMS) [62, 63] that link specific primers with fluorescent probes, selectively amplifies only DNA containing the target allele. SARMS detection methods targeted to *EGFR* mutations are used in two commercial assays (QIAGEN therascreen^®^ EGFR Rotor-Gene Q [RGQ] PCR kit [QIAGEN, Manchester, UK] and the Roche cobas^®^ EGFR mutation test v2 [Roche Molecular Systems, Inc., CA, USA]) that were approved by the US Food and Drug Administration (FDA) in 2013 as companion diagnostics to select patients suitable for EGFR TKI therapy [64]. In June 2016, the FDA approved the use of the Roche cobas^®^ EGFR mutation test v2 with plasma samples. Silencing of non-mutant DNA amplification using non-amplifiable PNA sequences, a synthetic DNA analog [65], directed against wild-type sequences (PNA clamping) has also been used to improve sensitivity. Such methods for ctDNA mutation analysis have demonstrated sensitivity relative to tumor averaging 65% [25]. Importantly, many of these methods have been shown to have high specificity ( > 88%), such that a positive result in plasma is highly likely to be linked to a positive result in the tumor [10, 25]. For example, a head-to-head comparison of the ability of the QIAGEN therascreen^®^ EGFR RGQ PCR kit *versus* PNA clamping to detect *EGFR* mutations in 96 patients with NSCLC revealed that specificity (100% for both) and sensitivity (65% for therascreen^®^ and 62% for PNA clamping) were high, and similar between assays [55]. In-house, laboratory-developed test solutions have also shown similar performance metrics and can be used, provided the appropriate level of performance has been demonstrated during assay validation.

Newer technologies with even greater sensitivity than existing assays may provide a means of reducing the false-negative rates observed with qPCR-based ctDNA testing methods. Methods based upon emulsion PCR, such as digital droplet PCR (ddPCR) and BEAMing [66] (Sysmex Inostics, Chou-Ku, Kobe, Japan) are enhancements of traditional PCR. These methodologies partition the sample into individual DNA fragments that are amplified in microdroplets, allowing scientists to detect mutant DNA even if only a few copies are present amongst a majority of wild-type DNA sequences. Such technologies have an analytical threshold as low as 0.001% of mutant DNA in a background of wild-type DNA [67]. However, as it becomes possible to detect extremely low levels of specific mutations, signals may be detected from small, subclinical clones that may not be relevant to treatment decisions at the time the sample is analyzed. This raises the question of whether the level of mutant DNA identified in the blood reflects the presence of a specific driver mutation within the primary tumor at a particular time. Whereas the threshold of detection of mutant DNA in liquid biopsy will lower with improvements in technology, it will not be easy to establish the clinical correlation between low levels of mutant DNA in plasma and the probability of response to targeted agents – further research is required to establish this. The fraction of mutant DNA in plasma depends on several variables, including: disease burden; the levels of expression of the mutation in the primary tumor; the rate of shedding of tumor DNA into the bloodstream; and the levels of DNA derived from non-transformed cells (inflammation of normal tissue surrounding the tumor, lysis of leukocytes after blood drawing etc). Such considerations are particularly relevant for detection of T790M, the most common resistance mechanism for first-line EGFR TKIs [68]. T790M mutations have sometimes been detected well in advance of any clinical progression using sensitive methods [11, 12]. However, determining when emergence of T790M mutations should be considered as indicators of ineffective TKI therapy, *versus* symptomatic progression, still remains to be resolved. Nevertheless, detection of T790M mutation at clinical progression of the disease using highly sensitive methods will enable patients to access a highly effective treatment.

While some PCR-based detection methods do not allow quantification of mutated gene copies, methods such as ddPCR and BEAMing enable absolute quantification of mutant *EGFR* levels in plasma and could be used for monitoring treatment response, disease progression, and early treatment failure associated with acquired drug resistance [69]. Robust data that link *EGFR* mutation levels detected in ctDNA to clinical endpoints are required to clarify what level of sensitivity is clinically relevant. With cut-offs set at 0.02% of mutant DNA, sensitivity of up to 86% and specificity of 98% *versus* tumor-based detection has been demonstrated for common activating *EGFR* mutations using the BEAMing platform [44, 70]. It is interesting to note that even these highly sensitive methods do not appear to detect *EGFR* mutations in the blood of every patient with a mutation in their primary tumor, indicating that there is a proportion of patients with NSCLC whose tumors do not shed sufficient amounts of DNA into the blood for molecular testing [32, 71]. It is likely that the most important factor when defining thresholds of sensitivity for technologies is the number of mutated ctDNA copies per volume of blood or plasma. Finally, it is important to underscore that different results were obtained by Thress et al when testing for the presence of T790M mutations in the plasma of patients experiencing disease progression following prior EGFR TKI therapy using the BEAMing and Roche cobas^®^ platforms [43]. These methods yielded a sensitivity of 73% and 81%, respectively, for the T790M mutation, although the corresponding specificities were lower at 67% and 58%, respectively. Of note, among 72 cases evaluated, 9 “false-positive” plasma results were detected by BEAMing, of which 7 tested positive using the Roche cobas^®^ test. Interestingly, the response rate to the “third-generation” TKI osimertinib (TAGRISSO^TM^ [AZD9291]) in patients with T790M-positive plasma samples (59%), was almost identical to that in patients with T790M-positive tissue samples (61%). Similar results were more recently obtained by Oxnard et al, in a large cohort of patients treated with osimertinib who underwent plasma *EGFR* mutation testing *via* BEAMing [70]. Altogether, these data suggest that plasma T790M mutation testing can be used to confirm whether the mutation is actually present in those patients whose tissue biopsy samples test negative for the mutation (likely due to heterogeneous disease), yet may actually benefit from TKI treatment targeting this resistance mutation.

In addition to the PCR-based method selected, it is the responsibility of the testing laboratory to implement basic measures to prevent contamination that may lead to false-positive results. The PCR reaction should be prepared in a laminar flow hood, with the pre- and post-PCR areas physically separated. Every PCR reaction must include the appropriate positive and negative controls, and it is recommended that negative samples are regularly (monthly, if possible) run in the laboratory from DNA extraction to PCR analysis, in order to ensure that there are no steps in the process in which contamination may occur.

### Next-generation sequencing and post-PCR methods

Next-generation sequencing (NGS) (otherwise known as massive parallel sequencing) describes a large group of technologies that can sequence millions of small DNA fragments in parallel. NGS shares some similarities with ddPCR, in that individual DNA fragments are isolated prior to sequencing, enabling detection of a small fraction of a mutated sequence. As such, NGS is a highly sensitive ctDNA solution providing a relative quantification (allelic frequency); with an accuracy comparable to that of ddPCR for ctDNA detection (99% accuracy) [72]. Of note, NGS techniques that include labelling of ctDNA fragments with barcode sequences (discriminating between reads of individual molecules to allow grouping of reads from each) have demonstrated near-complete elimination of false-positive results that can be applied to *EGFR* mutation analysis [73]. To date, limited ctDNA data are available on NGS platforms in NSCLC. A proof-of-concept study, on the use of NGS to screen ctDNA for a range of tumor biomarkers, found that between 68 matched tumor and ctDNA samples of patients with newly diagnosed metastatic NSCLC, overall mutation status concordance (including *BRAF, EGFR, ERBB2, KRAS*, and *PI3KCA* amplicons) was 68%, with a sensitivity of 58% and specificity of 87% [74]. Uchida et al conducted a prospective study to evaluate biopsy samples from 288 treatment-naïve patients with NSCLC using an NGS *EGFR* mutation detection system, utilizing genotyping results from biopsy samples as a comparator. Diagnostic sensitivity and specificity results were as follows: exon 19 deletion, 50.9% and 98.0%, respectively; L858R mutation, 51.9% and 94.1%, respectively. Uchida et al concluded that NGS “deep sequencing” of plasma DNA can be used for genotyping of *EGFR* in lung cancer patients, with the high specificity results in particular indicating that the system could enable a direct EGFR TKI recommendation on the basis of plasma-positive results [53]. In another study that aimed to detect sensitizing and resistance *EGFR* mutations in NSCLC using NGS of plasma ctDNA samples, results from the first five patients tested (from a cohort of 31 who had a repeat biopsy) indicated that multiplexed NGS is feasible and shows a high concordance with results from tumor biopsy [75]. Thress et al also utilized NGS to analyze ctDNA from seven patients with NSCLC who had developed resistance to osimertinib, and had an acquired *EGFR* C797S mutation detected in one sample [14]. A commercial laboratory based in the USA has used NGS to screen for a panel of mutations in the ctDNA of 1000 patients with cancer (including lung cancer), and detected ctDNA in 78% of these patients (of these, 74% had an actionable genetic aberration) [76, 77]. More recently, Rachiglio et al performed NGS analysis of ctDNA sampled from 44 patients with metastatic NSCLC. *EGFR* mutations were detected in 17/22 samples of patients with *EGFR* mutation-positive tumors (sensitivity 77%), as well as in samples of two patients whose tumors were not known to harbor *EGFR* mutations (both confirmed *via* ddPCR in plasma and tissue samples) [78]. Villaflor et al evaluated a targeted ctDNA NGS gene panel in 31 patients with NSCLC who provided matching tissue and blood samples: of 7 samples with an activating *EGFR* mutation detected in either tissue or blood, a concordance rate of 71% was calculated [79]. Yao et al performed targeted DNA sequencing to screen for driver gene mutations in matched ctDNA and tissue samples from 39 Chinese patients with NSCLC; the overall gene mutation concordance was 78.2% between matched samples, with sensitivity of *EGFR*, *KRAS*, *PIK3CA* mutations, and gene rearrangements detected in ctDNA at 70.6%, 75%, 50%, and 60%, respectively [54]. Chabon et al employed NGS (CAPP-Seq) to study resistance mechanisms of ctDNA samples of 43 patients with NSCLC treated with rocelitinib. In 28 patients, one or more putative resistance mechanisms were identified (of which the most common was MET copy-number gain as observed in 11 patients) [80].

The ability of NGS to analyze several genes in parallel has the potential to allow a single plasma sample to be used to determine eligibility for different specific therapies.

Methods employed after PCR amplification that have less commonly been used for *EGFR* mutation analysis in ctDNA samples of patients with NSCLC, and so are not discussed in detail here, include denaturing high-pressure liquid chromatography, mass spectrometry, and high-resolution melting analysis (reviewed by Huang et al [3]).

## CONCLUSIONS AND RECOMMENDATIONS

**Figure 2:**
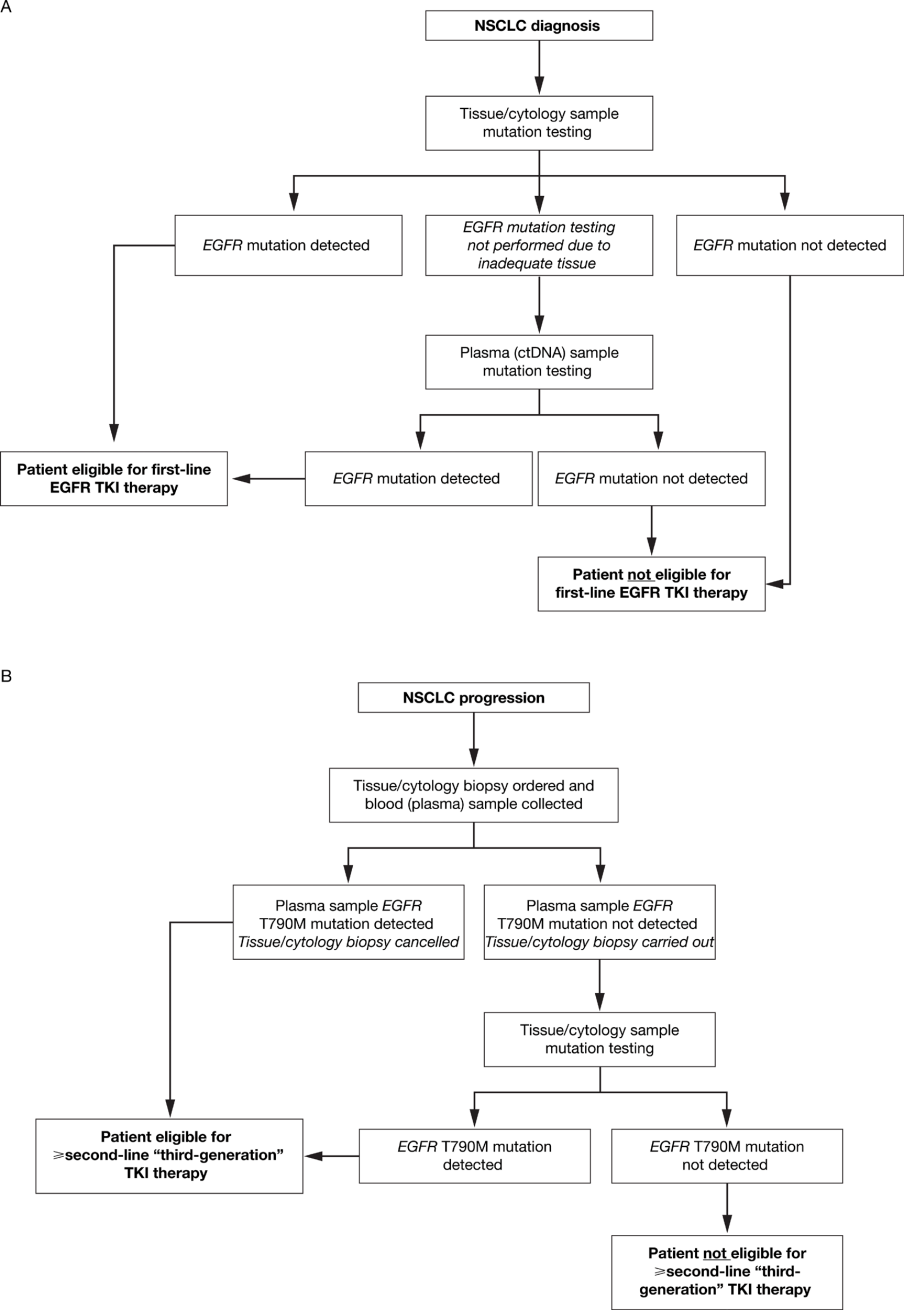
ctDNA mutation testing: recommended use to inform treatment selection at **A**. **NSCLC diagnosis and B. NSCLC progression following first-line TKI therapy**. At diagnosis of NSCLC **A**., plasma (ctDNA) sample testing is recommended when it is not possible to obtain adequate or suitable tissue at biopsy. In patients who have progressed following first-line TKI therapy **B**., plasma (ctDNA) and tissue/cytology sample testing is recommended to determine whether the T790M mutation is present, which informs eligibility for subsequent TKI therapy. ctDNA, circulating free tumor-derived DNA; EGFR, epidermal growth factor receptor; NSCLC, non-small-cell lung cancer; TKI, tyrosine kinase inhibitor.

Molecular testing for actionable mutation status information, including use of targeted treatments, has become routine practice across the field of clinical oncology [81]. Use of ctDNA to detect activating mutations provides a promising diagnostic approach if tumor tissue is not accessible. Another advantage of ctDNA is that mutant DNA from all tumors within a patient (including metastases) can be sampled, which may reduce the risk of missing a mutation due to tumor heterogeneity or sampling issues. The ASSESS study suggested that ctDNA use could detect *EGFR* mutations that may have been missed in the tumor due to use of cytology and small biopsies that can be problematic for *EGFR* analysis [27]. Of interest, additional analyses of ASSESS study data found that detection of *EGFR* mutations in plasma was significantly more likely when patients had a higher metastatic tumor burden and distant metastases [82]. In this respect, in patients with progressive disease the liquid biopsy may change to become positive over time with the increase in tumor burden. However, current evidence suggests that, despite using the latest, highly sensitive technologies, ctDNA does not allow detection of the common *EGFR* activating mutations in all patients with NSCLC and mutations in their tumors. For this reason, tumor tissue, where available, should be the first sample of choice for *EGFR* testing to provide an initial molecular diagnosis, to determine if patients are suitable for TKI therapy. However, in some cases, tissue test results are not evaluable (for example, due to low tumor cell count or degraded DNA) or tissue is not available [46, 83, 84]. ctDNA provides a viable alternative in this setting (Figure 2A). A recent clinical study of patients with NSCLC whose tissue samples could not be evaluated in mutation testing (*n* = 3) found that upon *EGFR* mutation detection in ctDNA samples, all presented with significant partial response to EGFR TKI therapy [85]. Interestingly, emerging data are demonstrating the feasibility of detecting *EGFR* mutations in ctDNA from urine samples, potentially providing a completely non-invasive alternative sample type in the absence of the preferred tumor tissue [86–88]. Furthermore, cerebrospinal fluid is also under investigation as a source of ctDNA for mutation analysis [89–92]; notably, *EGFR* mutations have been detected in ctDNA sampled from the cerebrospinal fluid of patients with lung adenocarcinoma and brain metastases [93]. Finally, the sensitivity of liquid biopsy could be increased by coupling the analysis of ctDNA and circulating tumor cells (CTCs) [94]. However, methods for CTC detection are complex, costly, and currently limited to the research setting.

When patients progress on first-line EGFR TKI therapy, and a new biopsy would otherwise be required to determine the molecular mechanism (eg T790M mutations) underlying the disease progression, ctDNA may have advantages over tissue. This is a particularly relevant subject, with a TKI targeting resistance mutations such as T790M [95, 96] recently approved in the USA, EU, and Japan (at time of writing). In fact, increasing evidence suggests that tumors are more heterogeneous following TKI therapy than at initial diagnosis [97]. In heterogeneous tumors, ctDNA samples may provide a better way to determine overall tumor mutation status than a small biopsy, which in most cases represents the available tissue sample at disease progression [94, 98]. Since ctDNA is much less invasive than a tissue biopsy, ctDNA may become the preferred option in this setting. However, due to the limited sensitivity of liquid biopsy, a new tissue sample should be obtained if the plasma test is negative for *EGFR* mutations (Figure 2B). Another advantage of using ctDNA is that it allows repeated sampling (monitoring) over time [99–104], with a faster mutation test turnaround time (TAT) compared with tissue [105] – something that could not be achieved using tumor biopsies. TAT is a relevant issue as it will directly impact upon the possibility of the patient to receive a targeted therapy. TAT may be influenced by several variables: the organization of the testing workflow; communication within the multidisciplinary team; the level of automation in the testing laboratory; and the mutation testing method employed. In our clinical experience, TAT tends to be quite short (3–4 days) for SARMS detection methods such as the QIAGEN therascreen^®^ EGFR RGQ PCR kit and the Roche cobas^®^ EGFR mutation test v2, and longer in more complex methods such as BEAMing (7–8 working days). Even greater TAT may be observed with NGS, perhaps between 10 and 14 working days, with bioinformatics needed for interpretation of the results.

Such studies can yield interesting information about how tumors evolve in response to treatment [11, 12, 106]; however, care must be taken not to over-interpret such findings. With the emergence of highly sensitive methods such as NGS and ddPCR, resistance mutations have been observed far in advance of clinical signs or radiographic progression. For T790M analysis, we recommend that ctDNA analysis should be performed at the same time as a tissue biopsy is ordered if available/feasible as each provides complementary information, and that monitoring should be restricted to clinical trials. Research should look to address remaining questions as to whether blood samples should be drawn at specific times of day, potentially based upon periods of fasting or exercise for example [107].

In this review, we have outlined the critical steps for obtaining an accurate result using ctDNA, including recommendations regarding standardization of plasma processing and handling, DNA extraction, and appropriate sensitive detection methods. In expert hands, good results can be obtained. In the real world, sensitivity and specificity have been observed to be lower than that achieved in centrally controlled clinical studies [41, 108], highlighting that standardization and training are vital to ensuring high-quality ctDNA testing in the clinic. Implementation of quality assurance schemes will be important to increase confidence in the quality of ctDNA testing outside of specialist laboratories. However, if well implemented, ctDNA has the potential to allow a great number of patients to benefit from targeted therapies.

## Abbreviations

AKT1: v-akt murine thymoma viral oncogene homolog 1
ARMS: amplified-refractory mutation system
AS: allele-specific
BRAF: B-Raf proto-oncogene, serine/threonine kinase
ctDNA: circulating free tumor-derived DNA
ddPCR: digital droplet polymerase chain reaction
EDTA: ethylenediaminetetraacetic acid
EGFR: epidermal growth factor receptor
ERBB2: erb-b2 receptor tyrosine kinase 2
ESR1: estrogen receptor 1
EQA: External Quality Assessment
FDA: US Food and Drug Administration
KRAS: Kirsten rat sarcoma viral oncogene homolog
LNA: locked nucleic acid
N/A: not available
NGS: next-generation sequencing
NSCLC: non-small-cell lung cancer
PCR: polymerase chain reaction
PIK3CA: phosphatidylinositol-4,5-bisphosphate 3-kinase catalytic subunit alpha
PME: polymer mediated enrichment
PNA: peptide nucleic acid
qPCR: quantitative polymerase chain reaction
RGQ: Rotor-Gene Q
SARMS: Scorpion amplified-refractory mutation system
TAT: turnaround time
TKI: tyrosine kinase inhibitor
